# Comparative effectiveness of tamoxifen, toremifene, letrozole, anastrozole, and exemestane on lipid profiles in breast cancer patients: A network meta-analysis

**DOI:** 10.1097/MD.0000000000018550

**Published:** 2020-01-10

**Authors:** Tao He, Wenhao Yang, Xinyi Zhang, Ping Li, Dandan Yang, Yunhao Wu, Yuan Fan, Mengya Xiang, Qianqian Huang, Jing Chen, Runke Zhou, Qing Lv, Jie Chen

**Affiliations:** a Department of Breast Surgery, West China Hospital/West China School of Medicine; b Department of Pediatrics, West China Second Hospital/West China School of Medicine; c West China School of Medicine, Sichuan University, Chengdu, Sichuan, China.

**Keywords:** breast cancer, endocrine therapy, lipids, meta-analysis

## Abstract

**Background:**

Adjuvant endocrine therapy is a vital portion of postoperative comprehensive treatment for breast cancer patients. In recent years, studies have shown that endocrine therapy has a certain impact on the serum lipids of breast cancer patients, and the changes of lipid profiles may bring a series of problems. However, very few studies focus on this issue to date. The results of these studies are inconsistent, and the influence of different adjuvant endocrine modalities on lipid profiles still remains controversial. In order to better explore this issue, we conduct this network meta-analysis.

**Method:**

The protocol followed preferred reporting items for systematic reviews and meta-analyses protocols. Three main databases (PubMed, Embase, and the Cochrane Library) will be searched systematically for eligible randomized controlled trials without language restriction. In addition, a manual search of the references of relevant published studies will also be considered. Two reviewers will conduct studies selection, data extraction, and risk of bias assessment independently. The primary outcome is the variation of biochemical parameters – the serum lipid profiles (cholesterol, triglyceride, high-density lipoprotein, low low-density lipoprotein).

**Results:**

The results will provide useful information about the side effects of different adjuvant endocrine drugs on lipid profiles in postoperative breast cancer patients (estrogen receptor-positive and/or progesterone receptor-positive).

**Conclusion:**

The findings of this study will be published in a peer-reviewed journal.

**Prospero Registration Number:**

CRD42019129850.

**Key Points**

(1) This network-meta analysis protocol poses a clearly formulated research question and methodology, to investigate a common side effect of endocrine drugs on lipid in breast cancer patients.
(2) Only randomized controlled trials will be included which may provide more unbiased results than other studies.
(3) The synthesis will be clearly structured according to an established cognitive theoretical framework.

## 1 Introduction

Breast cancer (BC) is one of the most common malignancies in women, with an incidence of approximately 2,100,000 new cases, and a mortality of approximately 630,000 cases worldwide in 2018.^[1]^ While, the mortality rates for BC patients have declined in the last decades due to early diagnosis and comprehensive treatments, thus leading to more BC survivors.^[2–4]^ Of note, those patients often die of other diseases (cardiovascular and cerebrovascular diseases, etc) rather than malignant tumors. With the increasing survival rate and the extension of life span in postoperative BC patients, it is quite necessary to pay enough attention to chronic diseases such as cardiovascular disease, dyslipidemia, hypertension, and so on.

Disease-free and overall survival have been improved in postoperative BC patients who were treated by endocrine therapy^[5–7]^; but several studies have reported that endocrine therapy had significant side effects on serum lipids in BC patients, which might offset the benefit of endocrine therapy.^[8–12]^ Subsequently, some researches further explore the effects of different endocrine therapy schemes on serum lipids; however, their findings are not consistent.^[13,14]^ Because of the shortage of head-to-head trials and the limitation of traditional pair-wise meta-analyses, the comparative influence of different endocrine drugs on serum lipid still remains controversial yet.

In this study, we attempt to perform a systematic review and network meta-analysis to compare the side effects of different endocrine drugs on serum lipid in postoperative BC patients who were estrogen receptor-positive and/or progesterone receptor-positive.

## 2 Methods

### 2.1 Registration

This study protocol has been registered in the PROSPERO, and the registration number is CRD42019129850. The protocol follows the preferred reporting items for systematic reviews and meta-analyses protocols (PRISMA-P) checklist and the PRISMA^[15]^ and Cochrane Handbook for Systematic Reviews of Interventions^[16]^ will be used as a guideline. This study is a meta-analysis of aggregate data which is no direct involvement with human subjects, thus ethical approval is waived.

### 2.2 Eligibility criteria

The detailed eligibility criterion are summarized using the PICOS approach (patients, intervention, comparisons, outcome, and study design type).

#### 2.2.1 Patients

Studies which contain patients who were surgically treated and pathologically diagnosed as BC with estrogen and/or progesterone receptor-positive will be included. There are no restrictions on age, ethnic distribution, and gender.

#### 2.2.2 Comparison of interventions

Postoperative BC patients will be treated with 1 of the following 5 endocrine drugs: tamoxifen, toremifene, letrozole, anastrozole, and exemestane. Trials which contain participants who do not receive endocrine therapy or receive other endocrine drugs will be excluded.

#### 2.2.3 Outcome measures

The primary outcomes are the variations of biochemical parameters: cholesterol (TC), triglyceride (TG), high-density lipoprotein (HDL), low low-density lipoprotein (LDL) which are serum lipids.

#### 2.2.4 Types of studies

Published randomized controlled trials (RCTs) with no language restriction up to April 11, 2019 will be included.

### 2.3 Patient and public involvement

There was no patient or public involvement in the development of this manuscript.

### 2.4 Search methods

PubMed, Embase, and the Cochrane Library will be systematically searched for eligible studies. The search strategy will involve terms including BC, tamoxifen, toremifene, letrozole, anastrozole, exemestane, lipids, and RCT. A detailed search strategy in PubMed, Embase, and the Cochrane Library is described in Table 1. Relevant studies and systematic reviews will also be scanned for additional eligible trials.

**Table 1:**
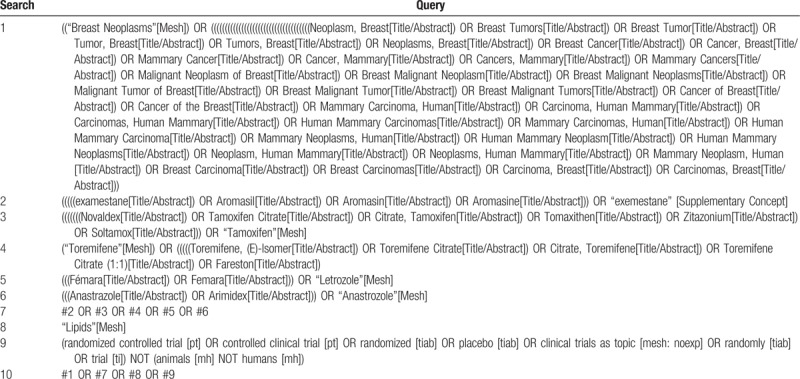
Preliminary search strategy in PubMed.

### 2.5 Study selection and data extraction

#### 2.5.1 Study selection

**Figure 1:**
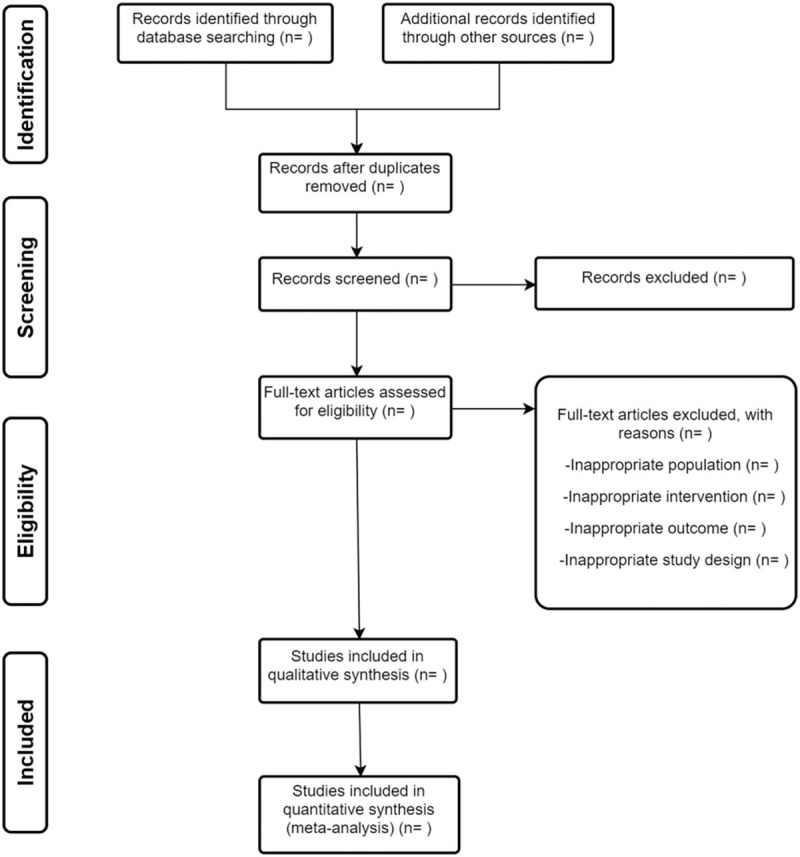
Flow diagram of study selection.

Study selection will be performed by 2 reviewers independently. The search results from 3 electronic databases and additional trials from other resources will be sent to Endnote. After duplicates removal, we will read the title and abstract to excluded most of the trials. Then, read full texts for further exclusion. The selection process will be summarized in a PRISMA flow diagram (Fig. 1). Any disagreements between the 2 authors should be resolved with the help of a third author.

#### 2.5.2 Data extraction

Two reviewers will perform data extraction independently. The data will include study characteristics, patient characteristics, data needed for quality assessment, and outcomes. Study characteristics include authors, countries, year of publication. Patient characteristics include type of interventions, age, sex, and diagnosis criteria. Outcomes include the variation of biochemical parameters, the serum lipid profiles (TC, TG, HDL, and LDL). All study characteristics will be summarized in the same standardized collection form. When extraction finished, data will be checked by the 2 reviewers. Any discrepancies should be resolved by negotiation between the 2 reviewers or a group discussion.

### 2.6 Risk of bias assessment

Two authors will independently assess the risk of bias of all included studies based on the Cochrane Collaboration's tool.^[17]^ The following contents will be evaluated: random sequence generation, allocation concealment, blinding of participants and researchers, blinding of outcome assessment, incomplete outcome data, selective reporting, and other biases. Each domain will be judged by the level of risk of bias: high level, low level, or unclear level. Any disagreements will be solved in group discussion.

#### 2.6.1 Pairwise meta-analyses

R software version 3.5.0 will be used to construct pairwise meta-analyses. Dichotomous data will be reported as risk ratios with their 95% confidence intervals (CIs). The mean difference and the 95% CI will be calculated for the continuous variable. *P* < .05 will be considered to be statistically significant. We will measure the heterogeneity of the included studies by *χ*^2^ and *I*^2^ test. If *χ*^2^ test with the significance set *P* > .10 and *I*^2^ < 50%, the heterogeneity is acceptable and a fixed effect model will be used for data analysis. Whereas a random-effects model will be used. Publication bias will be assessed by the Bayesian funnel plot and Egger regression if the study includes 10 or more studies.^[18,19]^

#### 2.6.2 Network meta-analyses

A Bayesian network meta-analysis will be performed with R x64 3.5.0. We will use the node splitting method to assess the inconsistency between direct and indirect comparisons if a loop exists.^[20]^ The surface under the cumulative ranking area values will be used to rank the different endocrine drugs.^[21]^ Comparison-adjusted funnel plots will be drawn to detect the small sample effects of on the results. A network plot will be conducted to present the comparisons of these 5 different endocrine drugs across trials to ensure if a network meta-analysis is feasible. Studies will be excluded if the drug investigated are not connected by other drugs. All the result figures will be generated using R x64 3.5.0 and STATA version 14.0 (College Station, TX).

#### 2.6.3 Confidence in cumulative evidence

The quality of evidence will be assessed based on the grading of recommendations assessment, development, and evaluation system. The evidence will be adjusted to 4 levels: high, moderate, low, or very low.

#### 2.6.4 Sensitivity and subgroup analysis

Sensitivity analysis will be carried out based on the sample size, the missing data result, and the methodological quality of the included studies. Subgroup analysis will be used to test relevant basis, premenopause, postmenopause, or age difference.

## 3 Discussion

Postoperatively, endocrine therapy is an important step in the comprehensive treatment of BC patients with estrogen receptor-positive and/or progesterone receptor-positive. Recently, more and more attention has been paid to the impact of endocrine therapy on blood lipid in patients with early BC. This is largely due to that the dyslipidemia caused by drugs might lead to a series of serious problems. However, a few studies have reported the side effect of endocrine drugs on serum lipids. Previous published RCTs, systematic review and head-to-head meta-analysis showed different conclusions on the impact of endocrine drugs on serum lipid, and thus, the comparative effectiveness of different drugs still remains controversial. Therefore, we conduct a network-meta analysis to investigate this question. To the best of our knowledge, this is the first network-meta analysis in this area. We aim to summarize direct and indirect evidence of published RCTs, and provide evidence-based suggestions for the clinical monitor of serum lipid during endocrine therapy.

## Author contributions

**Conceptualization:** Jie Chen, Tao He.

**Data curation:** Wenhao Yang, Xinyi Zhang.

**Methodology:** Ping Li, Dandan Yang, Yunhao Wu, Yuan Fan, Runke Zhou.

**Project administration:** Mengya Xiang, Qianqian Huang, Jing Chen.

**Supervision:** Jie Chen.

**Writing – original draft:** Tao He.

**Writing – review and editing:** Jie Chen, Tao He.
